# Exploring potential additive effects of 5-fluorouracil, thymoquinone, and coenzyme Q10 triple therapy on colon cancer cells in relation to glycolysis and redox status modulation

**DOI:** 10.1186/s43046-025-00261-7

**Published:** 2025-03-10

**Authors:** Akhmed Aslam, Faisal Minshawi, Hussain Almasmoum, Riyad Almaimani, Aiman Alsaegh, Amani A. Mahbub, Mohammad S. Iqbal, Aisha Tabassum, Mohamed E. Elzubier, Shakir Idris, Wesam F. Farrash, Bassem Refaat

**Affiliations:** 1 Department of Clinical Laboratory Sciences, Faculty of Applied Medical Sciences, Umm Al-Qura University, Makkah, Saudi Arabia; 2 Department of Biochemistry, Faculty of Medicine, Umm Al-Qura University, Makkah, Saudi Arabia; 3 Department of Pathology, Batterjee Medical College, Medicine Program, Jeddah, Saudi Arabia

**Keywords:** Chemoresistance, Cell cycle, Apoptosis, Oxidative stress, PI3K/AKT/mTOR pathway, Warburg effect

## Abstract

**Background:**

To investigate the anticancer effects of 5-Fluorouracil (5-FU), thymoquinone (TQ), and/or coenzyme Q10 (CQ10), alone and combined, in HT29, SW480, and SW620 human colorectal cancer (CRC) cell lines.

**Methods:**

Cell cycle progression and apoptosis were assessed by flow cytometry. Gene and protein expression of molecules involved in apoptosis (BLC2, survivin, BAX, Cytochrome-C, and Caspase-3), cell cycle (CCND1, CCND3, p21, and p27), the PI3K/AKT/mTOR/HIF1α oncogenic pathway, and glycolysis (LDHA, PDH, and PDHK1) were also analysed by quantitative RT-PCR and Western blot. Oxidative stress markers (ROS/RNS, MDA, and Protein carbonyl groups) and antioxidants (GSH and CAT) were quantified by ELISA.

**Results:**

All treatments resulted in anticancer effects depicted by cell cycle arrest and apoptosis, with TQ demonstrating greater efficacy than CQ10, both with and without 5-FU. However, 5-FU/TQ/CQ10 triple therapy exhibited the most potent pro-apoptotic activity in all cell lines, portrayed by the lowest levels of oncogenes (CCND1, CCND3, BCL2, and survivin) and the highest upregulation of tumour suppressors (p21, p27, BAX, Cytochrome-C, and Caspase-3). The triple therapy also showed the strongest suppression of the PI3K/AKT/mTOR/HIF1α pathway, with a concurrent increase in its endogenous inhibitors (PTEN and AMPKα) in all cell lines used. Additionally, the triple therapy favoured glucose oxidation by upregulating PDH, while decreasing LDHA and PDHK1 enzymes. The triple therapy also displayed the most significant decline in antioxidant levels and the highest increases in oxidative stress markers.

**Conclusions:**

This study is the first to demonstrate the superior anticancer effects of TQ compared to CQ10, with and without 5-FU, in CRC treatment. Moreover, this is the first report to reveal improved anticancer effects of the 5-FU/TQ/CQ10 triple therapy, potentially through promoting oxidative phosphorylation, attenuating the PI3K/AKT/mTOR/HIF1α pathway, and increasing oxidative stress-induced apoptosis.

**Graphical Abstract:**

Human colon cancer cells (HT29, SW480, & SW620) were treated with 5-Fluorouracil (5-FU), thymoquinone (TQ), and/or coenzyme Q10 (CQ10), individually and combined, for 12h. The anticancer effects related to cell cycle and apoptosis, expression of the PI3K/AKT/mTOR oncogenic pathway, glycolytic enzymes, and oxidative stress markers were measured. The triple therapy protocol revealed the best anticancer effects in all cell lines

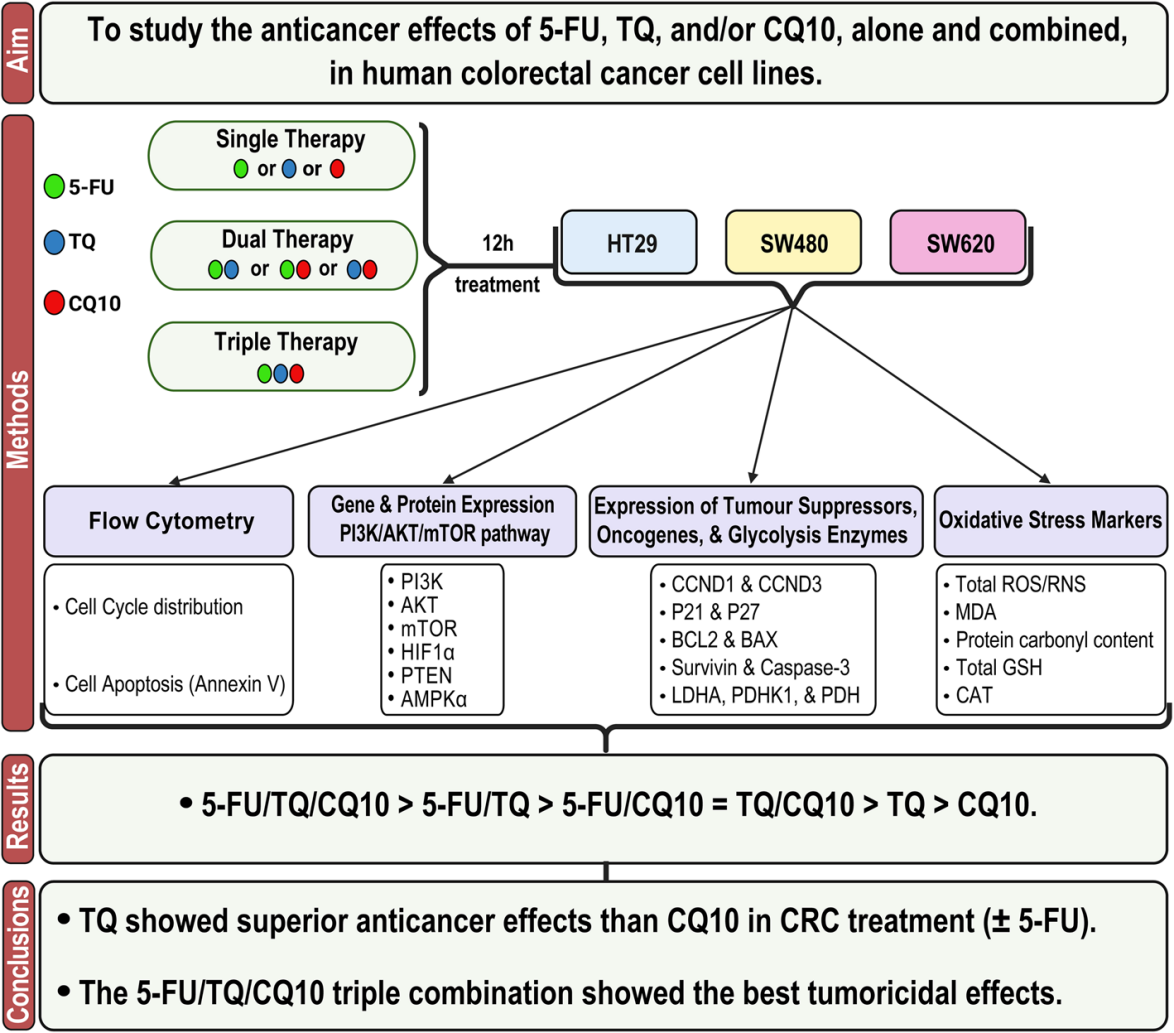

**Supplementary Information:**

The online version contains supplementary material available at 10.1186/s43046-025-00261-7.

## Background

Colorectal cancer (CRC) is a global health concern, being the third most prevalent malignancy and the fourth leading cause of cancer-related mortality [1, 2]. A hallmark of CRC is the overexpression of oncogenes such as cyclin D1 (CCND1), CCND3, and B-cell lymphoma 2 (BCL2), thereby promoting cell proliferation and inhibiting apoptosis [3, 4]. Concurrently, the downregulation of tumour suppressor genes, including cell cycle inhibitors (e.g., p21 & p27) and pro-apoptotic proteins like BCL2-associated X protein (BAX), cytochrome C (Cyto-C), and caspase-3 (Casp-3), further contributes to uncontrolled cell growth and survival [5–7].

The main chemotherapeutic agent for CRC, 5-Fluorouracil (5-FU), acts by inhibiting nucleic acid synthesis, thereby suppressing cancer cell proliferation [8, 9]. Moreover, 5-FU induces oxidative stress-mediated cell death through the production of reactive oxygen species (ROS) and reactive nitrogen species (RNS) [10, 11]. However, cancer cells develop chemoresistance by upregulating antioxidants [8, 9] and undergoing metabolic reprogramming, shifting towards aerobic glycolysis, a phenomena known as the Warburg effect [12–14].

This metabolic shift enables CRC cells to sustain proliferation and survival under therapeutic stress, by upregulating lactate dehydrogenase-A (LDHA) and suppressing pyruvate dehydrogenase (PDH) activity via increasing its inhibitory kinases (PDHKs) [12–14]. Notably, CRC cells further reinforce this metabolic adaptation by establishing an acidic tumour microenvironment, which has been shown to impair 5-FU uptake and cytotoxicity, exacerbating chemoresistance [10–14].

CRC cells also sustain resistance to 5-FU by hyper-stimulating the phosphatidylinositol-3-kinase (PI3K), which activates mammalian target of rapamycin (mTOR) via protein kinase B (AKT) [15, 16]. This molecular pathway is a key regulator of cell survival and metabolic adaptation [10–14], and its hyperactivation upregulates hypoxia-inducible factor-1α (HIF1α), promoting glycolytic reprogramming by enhancing LDHA expression and suppressing PDH activity [15–18]. Cancer cells further amplify PI3K/AKT/mTOR oncogenic pathway by inhibiting its natural suppressors, phosphatase and tensin homolog (PTEN) and 5' adenosine monophosphate-activated protein kinase-α (AMPKα) [15–18]. By sustaining metabolic adaptations and promoting tumour survival, these molecules contribute to chemoresistance development, highlighting the need for targeted therapies to overcome metabolic vulnerabilities during the treatment of CRC [10–14].

Numerous studies have emphasised the potential of nutraceutical compounds in CRC treatment [10, 19]. Thymoquinone (TQ), the main active ingredient of black seed, has shown promising anticancer effects against CRC and other malignancies by suppressing the PI3K/AKT/mTOR pathway, promoting oxidative glycolysis, increasing ROS levels, and inducing apoptosis [20–25]. Combining TQ with 5-FU has also demonstrated improved anti-tumorigenic effects in preclinical CRC studies [25–27]. On the other hand, coenzyme Q10 (CQ10), a vital component of the mitochondrial respiratory chain, is crucial for oxidative phosphorylation and maintaining redox homeostasis [28, 29]. Decreased serum levels of CQ10 have been linked to development and poorer prognosis of a variety of cancers [30–32]. CQ10 treatment has also been reported to suppress development and progression of various malignancies, both in vitro and in vivo [33–35], by inhibiting fermentative glycolysis, promoting glucose oxidation, and enhancing ROS-induced apoptosis [36–39]. Nevertheless, the anticancer effects of combining CQ10 with chemo or radiotherapy remain controversial, with some studies reporting improved cytotoxicity [40, 41], while others observed diminished efficacy [42, 43].

At present, the potential synergistic effects of combining CQ10 with either 5-FU or TQ in CRC treatment, as well as the comparative anticancer effects of TQ and CQ10, with and without chemotherapy, remain unexplored. Therefore, this research investigated the anticancer effects of 5-FU, TQ, and/or CQ10, alone and combined, on cell cycle progression, apoptosis, redox homeostasis, the expression of glucose metabolising enzymes, and the modulation of the PI3K/AKT/mTOR oncogenic pathway in human CRC cell lines.

## Materials and methods

### Cell culture techniques and therapeutic intervention regimens

All cell culture reagents were sourced from Thermo Fisher Scientific (MT, USA). Human HT29, SW480, and SW620 colon cancer cells (ATCC; VA, USA) were cultured in a humidified incubator (37°C and 5% CO_2_). These cell lines were chosen to represent the heterogeneity of CRC, with HT29 and SW480 modelling early-stage disease and SW620 representing metastatic CRC. This selection ensures a comprehensive evaluation of the therapeutic protocols across varying CRC stages and molecular profiles, enhancing the translational relevance of the findings. Specific media utilised were RPMI-1640 for HT29 and SW620 cells, and DMEM for SW480 cells. All media were supplemented with 10% foetal bovine serum (FBS) and 1% antibiotic–antimycotic solution.

5-FU (95% purity) was purchased from Hospira Australia Ltd. (Melbourne, Australia), whilst thymoquinone (TQ; 99.59% purity) and coenzyme Q10 (CQ; 98% purity) were from MedChemExpress LLC (NJ, USA). The IC50 concentrations of 5-FU, TQ, and CQ were determined using an MTT cytotoxicity assay after 24-h (24h) treatments (Suppl. Figure 1), as previously reported [25, 44]. Subsequently, the IC50 concentrations of each compound were utilised to evaluate the potential additive effects of combined therapeutic protocols while reducing the treatment duration to 12h [25, 44]. This approach preserves the effective IC50 concentrations of each individual compound for assessing synergistic or additive therapeutic interactions on cell cycle progression, apoptosis, and the gene and protein expression of targeted molecular pathways within a shorter treatment timeframe [25, 44]. Treatment with 5-FU (50 μM), TQ (75 μM), and/or CQ (100 μM) for 12 h established the following groups: untreated control (Ctr), monotherapy groups, TQ/5-FU (TF), CQ/5-FU (CF), TQ/CQ (TC) dual therapy groups, and TQ/CQ/5-FU (TCF) triple therapy group.

### Cell cycle analysis

HT29, SW480, and SW620 cells were prepared for cell cycle analysis by seeding in 6-well plates 24h before treatment initiation. Following trypsinisation and washing with PBS, fixation of cells was achieved by 24h incubation at 4°C in ice-cold 70% ethanol. Fixed cells were treated with RNase A (20 μg/ml, 15 min), washed, and stained with 2 µg/ml of propidium iodide (PI). An Acea Novocyte 3000 flow cytometer (Agilent Technologies, CA, USA) was used to determine percentage of cells in each cell cycle phase (20,000 events per sample; *n* = 3 per group). Data analysis was performed using the NovoExpress software cell cycle algorithm, as reported earlier [25, 44].

### Cell apoptosis

To assess treatment-induced cell death, the Annexin V-FITC/PI Apoptosis Assay Kit (Thermo Fisher Scientific) was utilised, as previously described [25, 44]. Briefly, HT29, SW480, and SW620 cells were harvested by trypsinisation, washed with ice-cold PBS (2x), and resuspended in 100 μl of 1 × Annexin V (AV) binding buffer. Cells were then stained by adding 5 μl of AV-FITC and 1 μl of PI stains per 100 μl cell suspension for a 15-min incubation period at room temperature in the dark. Subsequently, 400 μl of AV binding buffer were added to each sample prior to flow cytometric analysis using the Acea Novocyte 3000. The data are expressed as mean ± SD (*n* = 3) of the percentage of live (AV-/PI-), early apoptotic (AV + /PI-), late apoptotic (AV + /PI +), and dead (AV-/PI +) cells.

### Real-time reverse transcription polymerase chain reaction

PureLink™ RNA Mini Kit was used for total RNA isolation from each treatment group, whilst the high-capacity reverse transcription kit was utilised for synthesising cDNA, according to the manufacturer's instructions (Thermo Fisher Scientific). All cDNA samples were then diluted with RNase-free water followed by preparing triplicate reactions for each sample in a 10 μl reaction volume containing 3 μl diluted cDNA (25 ng), 1 μl of each gene-specific primer pair (Suppl. Table 1), and 5 μl of SYBR Green Master Mix (Thermo Fisher Scientific). Real-time PCR was then performed on a QuantStudio™ 3 System (Thermo Fisher Scientific) to quantify the relative expression levels of CCND1, CCND3, p21, p27, BCL2, Survivin, BAX, Cytochrome C, Caspase-3, PI3K, AKT1, mTOR, RAPTOR, RICTOR, PTEN, HIF1α, LDHA, PDH, and PDHK1. Negative controls included a no-template control, where cDNA was replaced with nuclease-free water, and a no-reverse-transcription control from the RT step. Gene expression normalisation was performed using GAPDH as the reference gene, and relative expression levels of the target genes were calculated using the 2^−∆∆Ct^ method, as described earlier [45].

### Protein expression by Western blotting

RIPA buffer with protease inhibitors (Thermo Fisher Scientific) was used to prepare total protein lysates from each cell pellet. Following electrophoretic separation in 4–20% SDS-PAGE gels (Bio-Rad Laboratories Inc.; CA, USA), the proteins were transferred to 0.2 μm PVDF membranes by a Trans-Blot® Turbo™ Transfer System (Bio-Rad Laboratories Inc.). After a 15-min blocking step with SuperBlock™ T20 buffer (Thermo Fisher Scientific), membranes were incubated overnight at 4°C with primary antibodies diluted 1:1000. While GAPDH, CCND3, Cytochrome C, and PI3K-p85α were detected by mouse monoclonal antibodies, rabbit monoclonal antibodies were used for CCND1, p21, p27, BCL2, BAX, cleaved Caspase-3, PTEN, AKT1, mTOR, LDHA, PDH, PDHK1, and HIF1α (Cell Signaling Technology Inc.; MA, USA). Following TBS-T washing, membranes were probed with 1:10,000 diluted anti-mouse or anti-rabbit WestVision™ peroxidase micropolymer secondary antibodies (Vector Laboratories Inc., CA, USA) for 1h at room temperature. SignalFire™ Plus ECL Reagent (Cell Signaling Technology) was used for signal detection followed by visualisation with a ChemiDocTM XRS + imaging system (Bio-Rad). Normalisation was performed using GAPDH loading control mouse monoclonal antibody (Thermo Fisher Scientific) and ImageJ software (https://imagej.net/ij/) was used for densitometric analysis, as previously described [25, 44].

### Markers of oxidative stress

Levels of antioxidants and markers of oxidative stress in cell lysates were assessed by commercial kits from Cell Biolabs (CA, USA). While quantification of total ROS/RNS was achieved by a fluorometric assay, malondialdehyde (MDA), protein carbonyl content (PCC), total glutathione (T-GSH), and catalase (CAT) concentrations were measured by colorimetric kits. Each cell lysate was analysed in duplicate according to manufacturer protocols following sonication in PBS (20 kHz for 3 s, applied in three pulses with 10-s intervals). Determination of fluorescence intensity (480 nm excitation/530 nm emission) and absorbance at varying wavelengths (T-GSH: 405 nm, MDA & PCC: 450 nm, and CAT: 520 nm) was achieved by a SpectraMax M2 Microplate Reader (Molecular Devices, CA, USA).

### Statistical analysis

Statistical analysis was performed using version 25 of SPSS software. The Kolmogorov–Smirnov test was used to evaluate data normality, whilst assessment of homogeneity of variance was by Levene's test. Depending on the equality of variance, Tukey's (for equal variances) or Games-Howell (for unequal variances) post-hoc tests following one-way ANOVA were employed for comparisons between groups. Statistical significance was established at a *P*-value of < 0.05.

## Results

### Effects of treatment protocols on cell cycle progression and expression of cell cycle-regulatory molecules

**Fig. 1:**
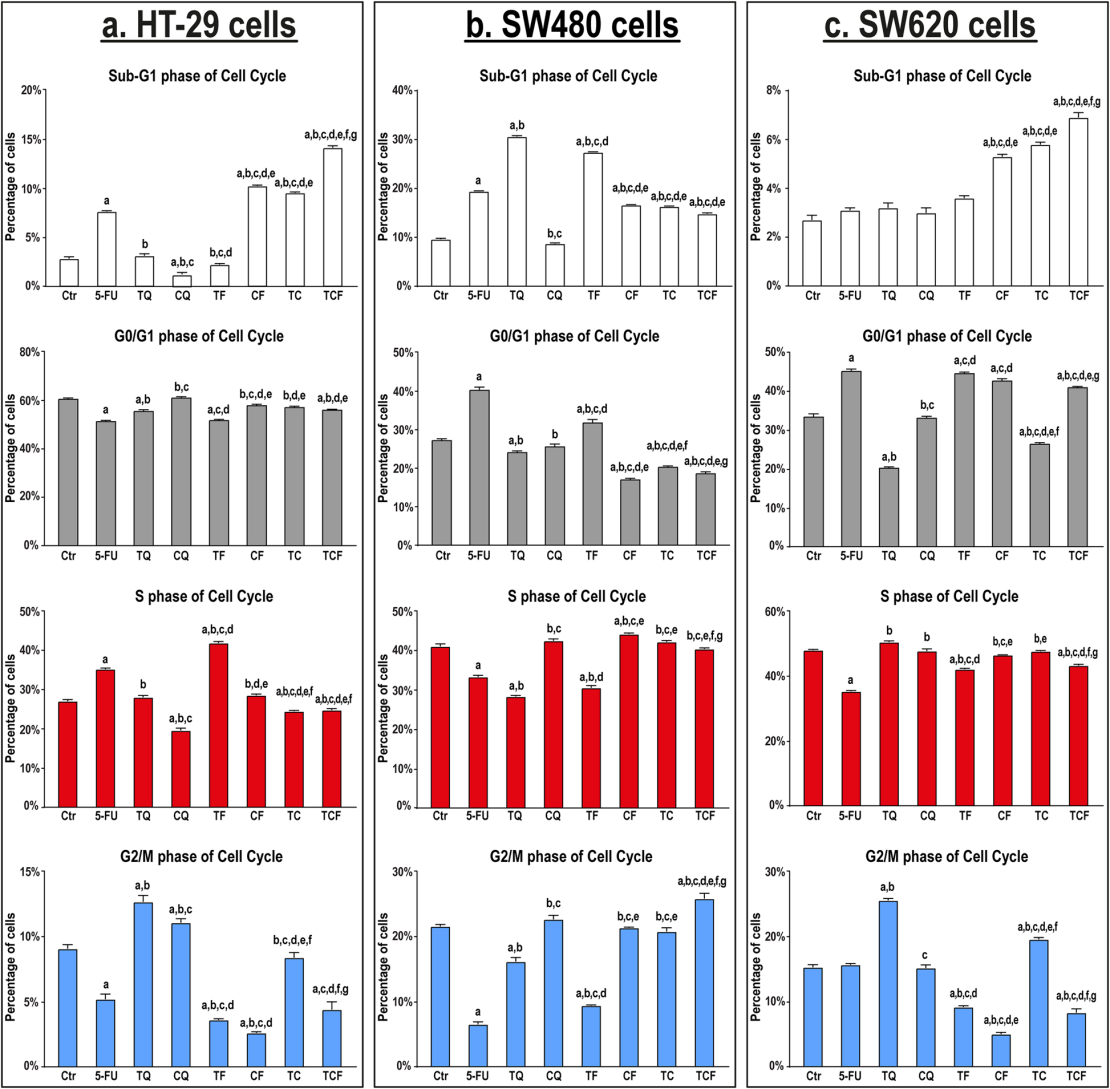
Percentage (mean ± SD) of (**a**) HT29, (**b**) SW480, and (**c**) SW620 CRC cells in different cell cycle phases after 12h treatment with 5-Fluorouracil (5-FU), thymoquinone (TQ), and/or coenzyme Q10 (CQ) as single agents, dual combinations (TF, CF, TC), or triple therapy (TCF). Data were analysed by one-way ANOVA followed by Tukey’s post-hoc test. Statistical significance: a = *P* < 0.05 compared to untreated cells; b = *P* < 0.05 compared to 5-FU group; c = *P* < 0.05 compared to TQ group, d = *P* < 0.05 compared to CQ group; e = *P* < 0.05 compared to TF group; f = *P* < 0.05 compared to CF group, and g = *P* < 0.05 compared to TC group)

Numbers of HT29 cells in the sub-G1 phase increased substantially with 5-FU monotherapy (2.7-fold), CF and TC dual therapies (3.6-fold & 3.4-fold, respectively), and TCF triple therapy (fivefold) compared to untreated controls (Fig. 1a). Marked elevations in the sub-G1 SW480 cell population were also observed following 5-FU and TQ monotherapies (twofold & 3.2-fold, respectively), TF, CF, and TC dual therapies (2.9-fold, 1.7-fold, & 1.7-fold, respectively), and TCF triple therapy (1.5-fold; Fig. 1b). In contrast, SW620 sub-G1 cell counts were equal between all monotherapies and non-treated cells, whilst the percentage increased significantly with CF (twofold), TC (2.1-fold), and TCF (2.6-fold) therapies (Fig. 1c).

HT29 cell population increased markedly in S-phase with both 5-FU single therapy (1.3-fold) and TF co-therapy (1.5-fold), whilst both TQ (1.4-fold) and CQ (1.2-fold) monotherapies were associated with cell cycle arrest at G2/M-phase (Fig. 1a). Although SW480 cells primarily displayed G0/G1-phase cell cycle arrest in response to 5-FU single (1.5-fold) and TF dual (1.2-fold) therapies, arrests at S-phase and G2/M-phase were observed with CF dual (1.1-fold) and TCF triple (1.2-fold) therapies, respectively (Fig. 1b). Similarly, SW620 cell percentage in G0/G1-phase increased markedly with 5-FU monotherapy (1.4-fold), TF and CF co-therapies (1.3-fold for both), and TCF triple therapy (1.2-fold), whereas TQ monotherapy (1.7-fold) and TC co-therapy (1.3-fold) induced G2/M arrest, compared with non-treated cells (Fig. 1c). The histogram plots displaying the cell cycle analysis for the HT29, SW480, and SW620 cell lines by flow cytometry are presented in Supplementary Figure 2.

**Fig. 2:**
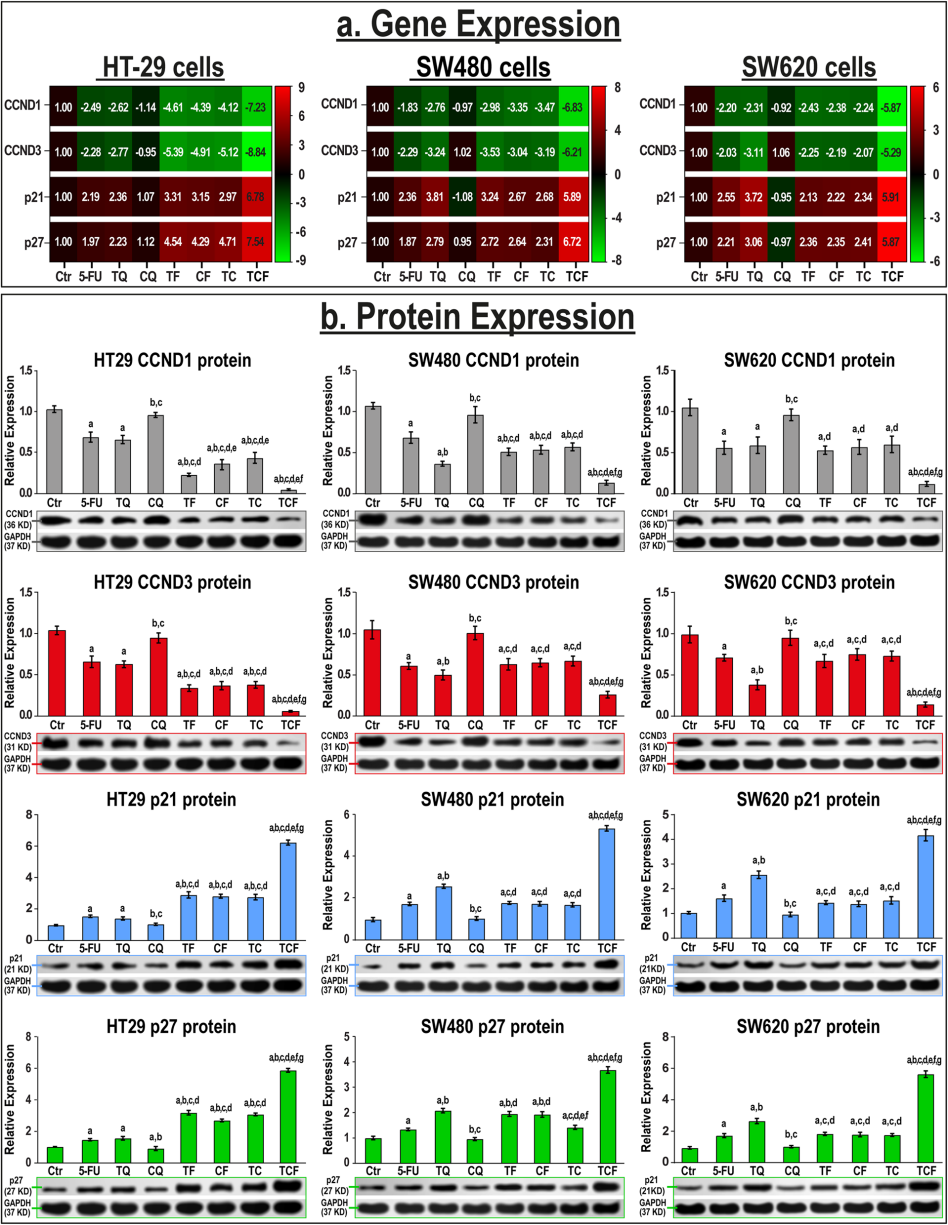
(**a**) Heatmap of relative mRNA expression (mean ± SD) of CCND1, CCND3, p21, and p27 genes, and (**b**) corresponding protein expression levels by Western blot after 12h treatment with 5-FU, TQ, and/or CQ as single, dual, or triple therapies in HT29, SW480, and SW620 CRC cells. Data were analysed by one-way ANOVA followed by Games-Howell post-hoc test. Statistical significance: a = *P* < 0.05 compared to untreated cells; b = *P* < 0.05 compared to 5-FU group; c = *P* < 0.05 compared to TQ group; d = *P* < 0.05 compared to CQ group; e = *P* < 0.05 compared to TF group; f = *P* < 0.05 compared to CF group; and g = *P* < 0.05 compared to TC group)

Furthermore, single treatment with 5-FU and TQ, but not CQ, markedly suppressed CCND1 and CCND3 gene and protein expression, whilst upregulating p21 and p27 compared to untreated HT29, SW480, and SW620 cells (Fig. 2). While 5-FU and TQ monotherapies resulted in similar expression profiles of the targeted cell cycle regulators in HT29 cells, TQ monotherapy in SW480 and SW620 cells showed significantly lower CCND1 and CCND3 with higher p21 and p27 expression relative to 5-FU group (*P* < 0.05 for all markers). Although all dual therapies equally reduced CCND1 and CCND3 expression, whilst increasing p21 and p27 mRNAs and proteins compared to all monotherapies in HT29 cells, their effects were equal to 5-FU monotherapy in SW480 and SW620 cell lines (Fig. 2). In contrast, the lowest CCND1 and CCND3 alongside the highest p21 and p27 gene and protein expression in all cell lines was observed with TCF triple therapy relative to all treatment groups (Fig. 2).

### Effects of treatment protocols on cell apoptosis and expression of markers of cell survival and cell death

**Fig. 3:**
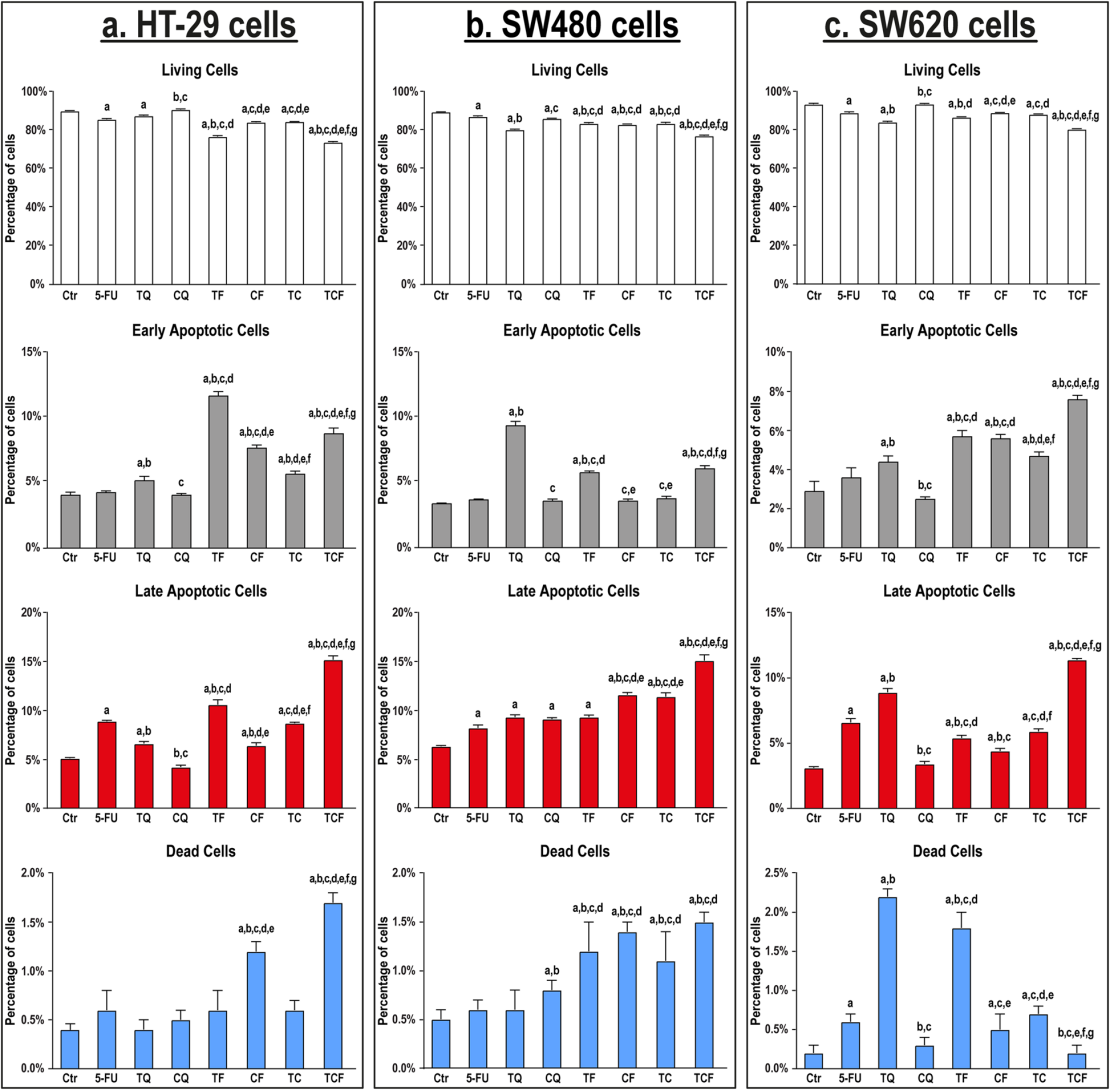
Percentage (mean ± SD) of live (AV-/PI-), early apoptotic (AV + /PI-), late apoptotic (AV + /PI +), and dead (AV-/PI +) cells after 12h treatment with 5-FU, TQ, and/or CQ in (**a**) HT29, (**b**) SW480, and (**c**) SW620 CRC cells. Data were analysed by one-way ANOVA followed by Tukey’s post-hoc test. Statistical significance: a = *P* < 0.05 compared to untreated cells; b = *P* < 0.05 compared to 5-FU group; c = *P* < 0.05 compared to TQ group; d = *P* < 0.05 compared to CQ group; e = *P* < 0.05 compared to TF group; f = *P* < 0.05 compared to CF group; and g = *P* < 0.05 compared to TC group)

Single treatment with 5-FU and TQ significantly reduced viable cell numbers in all cell line tested (Fig. 3). While both monotherapies decreased HT29 cell viability equally (1.1-fold for both drugs), TQ single therapy tended to be more effective in SW480 and SW620 cell lines (1.1-fold for both cell lines; *P* < 0.05 in both cell lines; Fig. 3). In contrast, CQ monotherapy selectively reduced viable SW480 cells (1.04-fold) relative to untreated cells and the effects were equal to 5-FU single therapy, but less effective than TQ monotherapy (Fig. 3). Moreover, all dual treatment protocols increased the percentage of non-viable cells, with the TF protocol displaying the greatest apoptosis across all cell lines used (Fig. 3). In HT29 (Fig. 3a) and SW620 (Fig. 3c), TF regimen exhibited the highest percentage of early (2.9-fold and twofold, respectively) and late (2.1-fold and 1.7-fold, respectively) apoptotic cells relative to untreated, single, and the other dual therapies. On the other hand, TF co-therapy induced the highest early apoptotic SW480 cells (1.7-fold), whereas CF and TC equally showed the highest late apoptosis (1.8-fold for both) relative to untreated cells and all monotherapies (Fig. 3b). While the triple therapy demonstrated the highest numbers of non-living cells in all cell lines tested, it induced the most increases in late apoptosis and cell death in HT29 (threefold & 4.3-fold, respectively; Fig. 3a) and SW480 (threefold & 4.3-fold, respectively; Fig. 3b) cell lines. In contrast, TCF displayed the maximal percentage of early (2.6-fold) and late (3.7-fold) apoptosis in SW620 cells compared with all other groups (Fig. 3c). The Scatter plots displaying the apoptosis analysis for the HT29, SW480, and SW620 cell lines by flow cytometry are shown in Supplementary Figure 3.

**Fig. 4:**
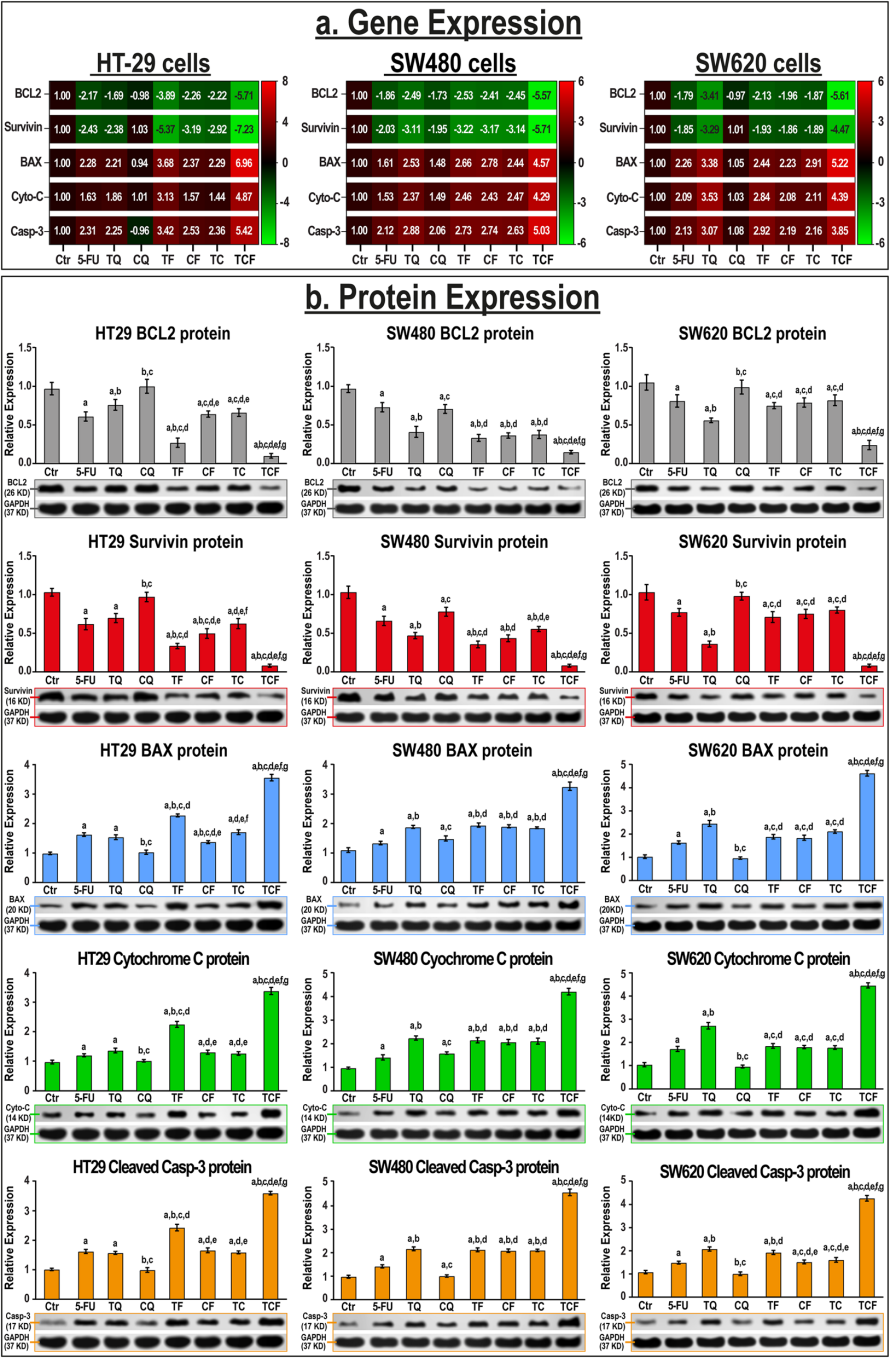
(**a**) Heatmap of relative mRNA expression (mean ± SD) of BCL2, survivin, BAX, cytochrome C, and caspase-3 genes, and (**b**) corresponding protein expression levels by Western blot after 12h treatment with 5-FU, TQ, and/or CQ in HT29, SW480, and SW620 CRC cells. Data were analysed by one-way ANOVA followed by Games-Howell post-hoc test. Statistical significance: a = *P* < 0.05 compared to untreated cells; b = *P* < 0.05 compared to 5-FU group; c = *P* < 0.05 compared to TQ group; d = *P* < 0.05 compared to CQ group; e = *P* < 0.05 compared to TF group; f = *P* < 0.05 compared to CF group; and g = *P* < 0.05 compared to TC group)

Additionally, both 5-FU and TQ monotherapies, but not CQ, exhibited marked reductions in BCL2 and survivin with increases in BAX, Cyto-C, and Casp-3 at the gene and protein levels in all cell lines relative to untreated cells (Fig. 4). Although these effects were stronger with 5-FU in HT29 cells (Fig. 4a), TQ monotherapy appeared more effective in SW480 (Fig. 4b; *P* < 0.05) and SW620 (Fig. 4c; *P* < 0.05) cells. Moreover, all dual treatment groups exhibited additional declines in BCL2 and survivin alongside further increases in BAX, Cyto-C, and Casp-3 mRNAs and proteins compared to all monotherapies in all cell lines used. Specifically, TF protocol showed the most effective effects in HT29 cells (Fig. 4a), whilst all dual therapies exhibited similar effects in SW480 (Fig. 4b) and SW620 (Fig. 4c) cells. On the other hand, the triple therapy regimen induced the strongest modulation of the gene and protein expression of cell survival and apoptosis markers in all cell line tested (Fig. 4).

### Gene and protein expression of the PI3K/AKT/mTOR tumorigenic pathway

**Fig. 5:**
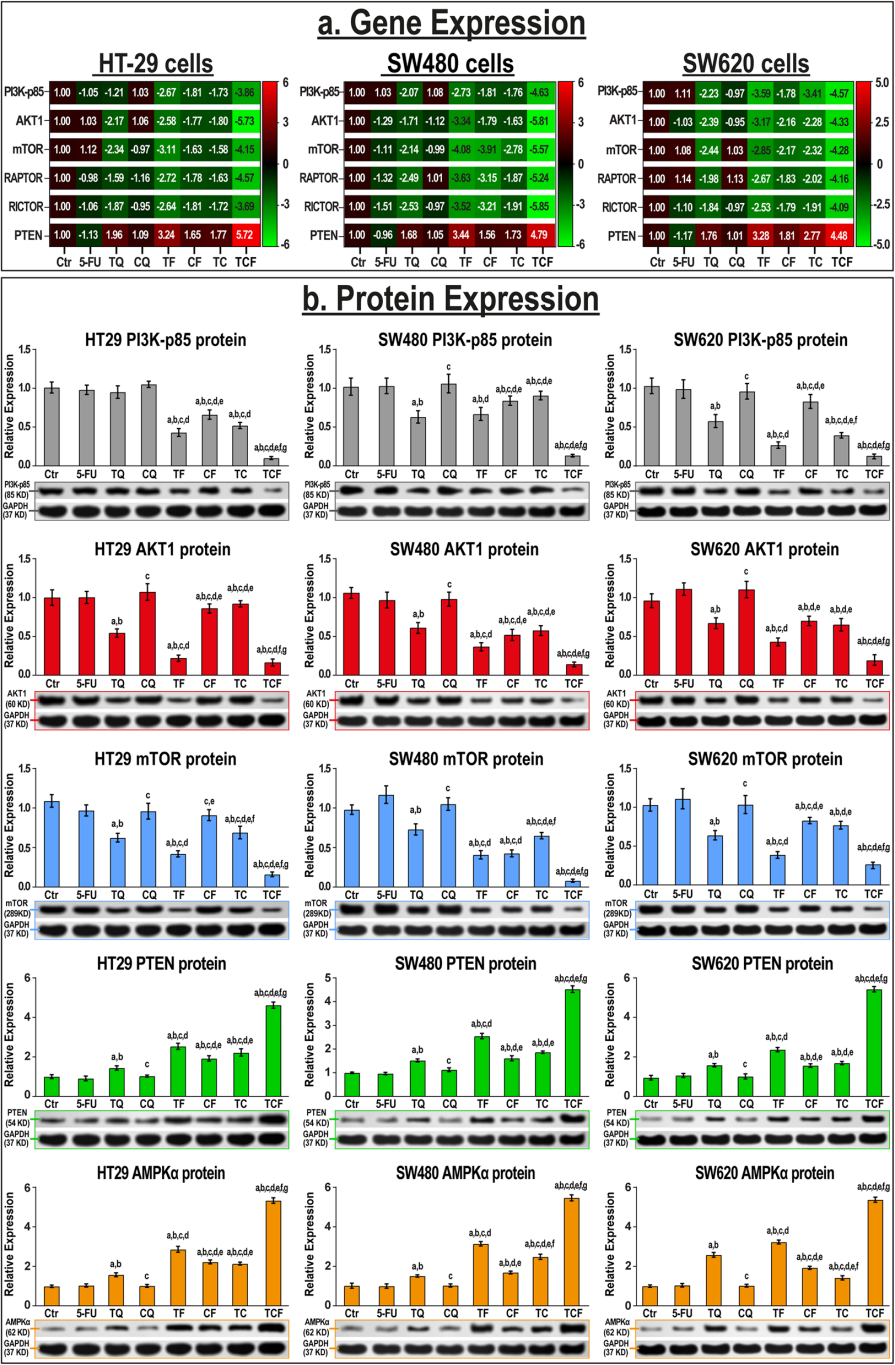
(**a**) Heatmap of relative mRNA expression (mean ± SD) of PI3K-p85, PTEN, AKT1, RAPTOR, RICTOR, and mTOR genes, and (**b**) relative protein expression levels by Western blot for PI3K-p85, PTEN, AKT1, mTOR, and AMPKα after 12h treatment with 5-FU, TQ, and/or CQ in HT29, SW480, and SW620 CRC cells. Data were analysed by one-way ANOVA followed by Games-Howell post-hoc test. Statistical significance: a = *P* < 0.05 compared to untreated cells; b = *P* < 0.05 compared to 5-FU group; c = *P* < 0.05 compared to TQ group; d = *P* < 0.05 compared to CQ group; e = *P* < 0.05 compared to TF group; f = *P* < 0.05 compared to CF group; and g = *P* < 0.05 compared to TC group)

Among monotherapies, only TQ exhibited marked declines in PI3K, AKT, mTOR, RAPTOR, and RICTOR gene expression and significantly increased PTEN mRNA compared to non-treated group in all cell line used (Fig. 5a). TQ monotherapy also significantly reduced PI3K, AKT, and mTOR, whilst upregulating PTEN and AMPKα proteins relative to untreated cells and other monotherapy groups across all cell lines tested (Fig. 5b; *P* < 0.05 for all markers). While all dual therapies further downregulated PI3K, AKT, mTOR, RAPTOR, and RICTOR alongside upregulated PTEN and AMPKα genes and proteins, these effects were more pronounced with the TF protocol compared to untreated, single, and the other dual therapies in all cell lines (Fig. 5). Nonetheless, the tiple therapy resulted in the strongest modulation of PI3K, AKT, mTOR, RAPTOR, RICTOR, PTEN and AMPKα mRNAs and proteins across all cell lines (Fig. 5).

### Gene and protein expression of glycolysis-regulatory molecules

**Fig. 6:**
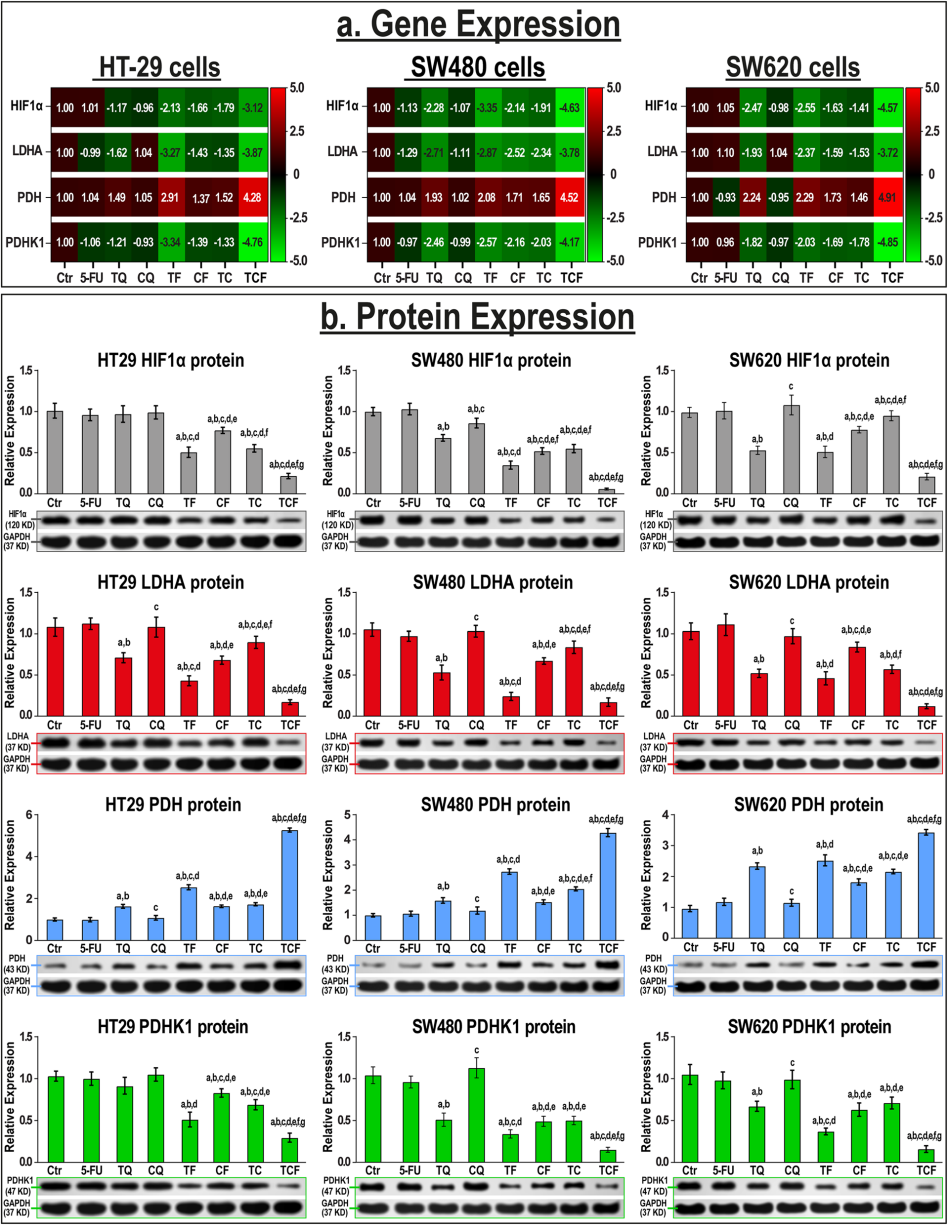
(**a**) Heatmap of relative mRNA expression (mean ± SD) of HIF1α, LDHA, PDH, and PDHK1 genes, and (**b**) relative protein expression levels by Western blot after 12h treatment with 5-FU, TQ, and/or CQ in HT29, SW480, and SW620 CRC cells. Data were analysed by one-way ANOVA followed by Games-Howell post-hoc test. Statistical significance: a = *P* < 0.05 compared to untreated cells; b = *P* < 0.05 compared to 5-FU group; c = *P* < 0.05 compared to TQ group; d = *P* < 0.05 compared to CQ group; e = *P* < 0.05 compared to TF group; f = *P* < 0.05 compared to CF group; and g = *P* < 0.05 compared to TC group)

TQ-alone, but not 5-FU and CQ, was associated with substantial declines in HIF1α, LDHA, and PDHK1, whilst increasing PDH enzyme, at the gene and protein levels compared to non-treated cells across all cell lines (Fig. 6; *P* < 0.05 for all markers). All co-therapy regimens further downregulated the gene and protein expression of HIF1α, LDHA, and PDHK1 alongside upregulated PDH in all cell lines, with TF dual therapy exhibiting the most significant modulatory effects. However, the triple therapy protocol demonstrated the lowest HIF1α, LDHA, and PDHK1 gene and protein expression with the highest upregulation in PDH mRNA and protein compared to all other treatments in all cell lines tested (Fig. 6).

### Levels of antioxidants and oxidative stress makers

**Fig. 7:**
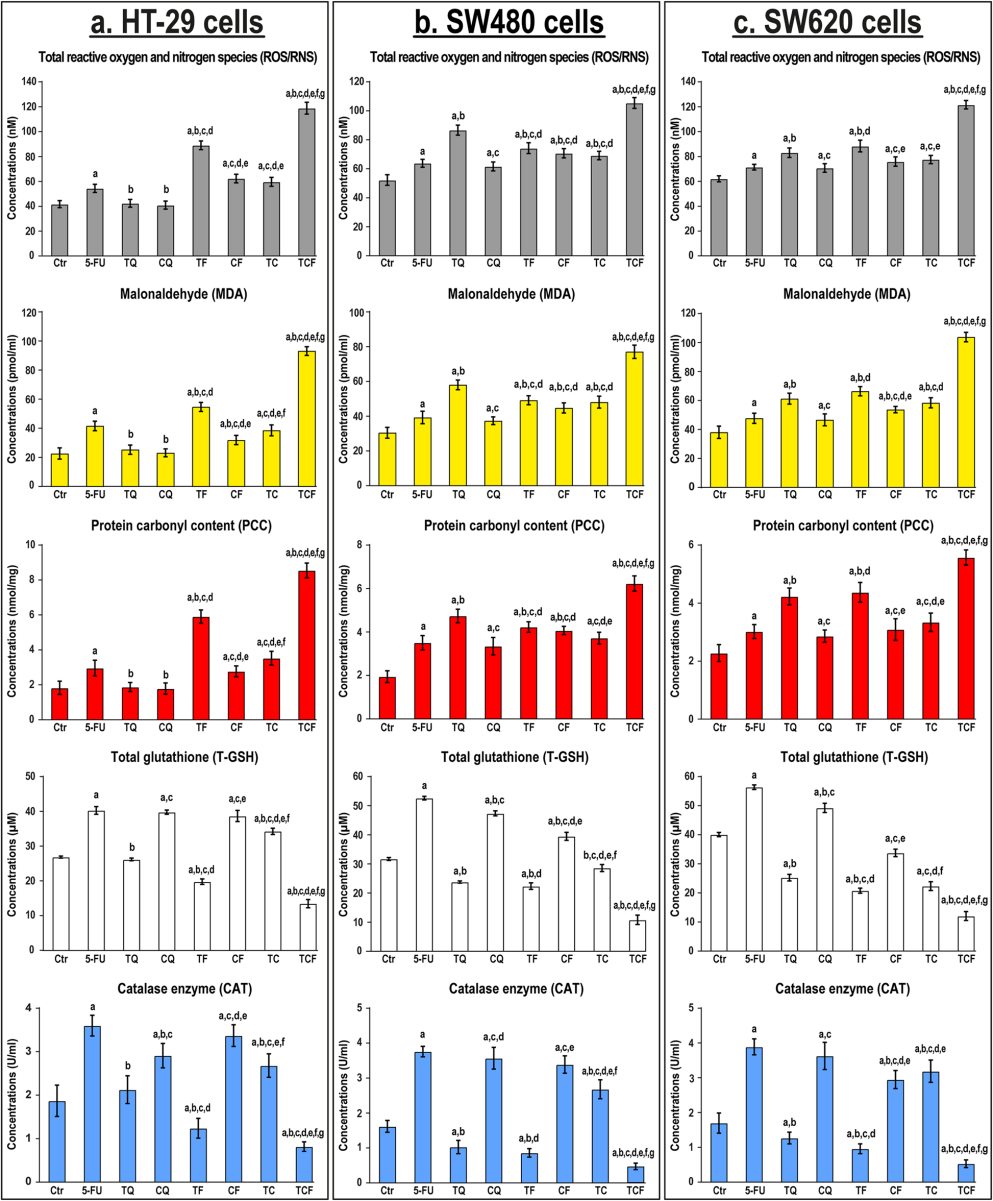
Concentrations (mean ± SD) of total reactive oxygen and nitrogen species (ROS/RNS), malondialdehyde (MDA), protein carbonyl content (PCC), total glutathione (T-GSH), and catalase (CAT) in cell lysates after 12h treatment with 5-FU, TQ, and/or CQ in (**a**) HT29, (**b**) SW480, and (**c**) SW620 CRC cells. Data were analysed by one-way ANOVA followed by Tukey’s post-hoc test. Statistical significance: a = *P* < 0.05 compared to untreated cells; b = *P* < 0.05 compared to 5-FU group; c = *P* < 0.05 compared to TQ group; d = *P* < 0.05 compared to CQ group; e = *P* < 0.05 compared to TF group; f = *P* < 0.05 compared to CF group; and g = *P* < 0.05 compared to TC group)

Single treatment with 5-FU and CQ equally and significantly elevated oxidative stress markers (ROS/RNS, MDA, & PCC) and antioxidants (total GSH & catalase enzyme) in all cell lines tested, compared to untreated controls (Fig. 7). While TQ displayed similar effects to the other monotherapies on pro-oxidants in HT29 cells, antioxidant levels were markedly lower than 5-FU and CQ protocols (Fig. 7a; *P* < 0.05 for all markers). However, TQ further increased ROS/RNS, MDA, and PCC levels while significantly reducing total GSH and catalase enzyme compared to non-treated controls and the other monotherapies in SW480 (Fig. 7b; *P* < 0.05 for all markers) and SW620 (Fig. 7c; *P* < 0.05 for all markers) cells. Markers of oxidative stress increased substantially, whilst antioxidants decreased with all dual therapies, with TF exhibiting more pronounced effects compared to untreated, single, and the other dual therapies in all cell lines (Fig. 7). On the other hand, the triple therapy was linked with the strongest modulation of oxidative stress markers and antioxidants across all cell lines used (Fig. 7).

## Discussion

This study investigated the synergistic potential of combining TQ and/or CQ10 with 5-FU to enhance its cytotoxic effects against CRC, with a focus on their effect on the PI3K/AKT/mTOR pathway, glycolysis regulatory enzymes, and redox homeostasis, key contributors to chemoresistance [12, 13, 46].

Our findings revealed similar IC50 values for 5-FU across all three CRC cell lines (HT29, SW480, and SW620), aligning with prior studies reporting consistent IC50 values for 5-FU at 48 h in HT29 and SW480 (19.3 µM and 17.5 µM, respectively) [47], and ~ 50 µM across multiple CRC cell lines following 72-h treatment [48]. Moreover, our data align with previous reports showing that 5-FU monotherapy induces cell cycle arrest by downregulating CCND1 and CCND3 while upregulating p21 and p27 [49–51]. Additionally, 5-FU triggered apoptosis via BCL2 and survivin downregulation, coupled with BAX, cytochrome C, and Caspase-3 upregulation, in agreement with prior research [52, 53]. The observed increase in ROS/RNS, MDA, and PCC following 5-FU treatment supports its role in ROS-mediated apoptotic cell death, as previously suggested [8, 9].

However, 5-FU resistance remains a major challenge in CRC treatment, as cancer cells evade ROS-induced apoptosis by elevating antioxidant defences [8, 9]. Moreover, they enhance chemoresistance by shifting towards aerobic glycolysis (Warburg effect) [13, 46], which involves LDHA upregulation and PDH inhibition via PDHK1 overexpression [17, 18]. This metabolic shift creates an acidic tumour microenvironment, impairing both 5-FU uptake and cytotoxicity [17, 18]. Additionally, cancer cells hyperactivate the PI3K/AKT/mTOR pathway by suppressing PTEN and AMPKα, leading to increased HIF1α expression, which further drives glycolytic reprogramming by boosting LDHA activity and inhibiting PDH [15–18].

In this study, 5-FU monotherapy increased antioxidant levels (GSH and CAT) in all CRC cell lines compared to untreated controls. However, the expression of PI3K/AKT/mTOR pathway components and glycolysis regulators remained unchanged between 5-FU-treated and non-treated groups across all cell lines. These findings suggest that cancer cells may initially resist 5-FU cytotoxicity by strengthening their antioxidant defences, while a shift towards glycolysis could be a later-stage adaptation for sustained chemoresistance [8, 9, 14–18]. However, more studies using varying 5-FU doses and longer treatment durations are needed to verify our hypothesis.

Several nutraceuticals have been proposed as potential effective therapies for CRC, including TQ [54–56] and CQ10 [33–35], due to their ability to modulate cellular metabolism and redox status. Our results demonstrated that TQ showed consistent IC50 value across the different cell lines used, which aligns with earlier studies revealing similar IC50 values for TQ in CRC cell lines, including HCT116 and SW480 at 24h [24], as well as in HCT116 and HT29 at 48h [57]. While none of the previous studies measured the IC50 values of CQ10 across multiple CRC cell lines, an earlier report revealed that the CQ10 analogue antroquinonol exhibited IC50 values of 18.6 µM in PANC-1 and 20.2 µM in AsPC-1 in pancreatic cancer cells [57]. Another CQ10 analogue, MitoQ, also demonstrated almost identical IC50 values of 0.33 µM in PANC-1 and 0.39 µM MiaPaCa-2 cells [58].

To our knowledge, this is the first study to directly compare the anticancer efficacies of TQ and CQ10, both alone and combined with chemotherapy. The findings of this study align with previous reports highlighting the anticancer properties of TQ [54–56], which include inducing cell cycle arrest and apoptosis in CRC cells by attenuating the PI3K/AKT/mTOR pathway, inhibiting HIF1α and LDHA, and promoting oxidative stress [20–24]. Additionally, our results support existing evidence that combining 5-FU with TQ enhances anticancer effects in CRC treatment [25–27].

In contrast, our data suggest that CQ10 is a less effective therapeutic agent against CRC, likely due to its strong antioxidant activity [28–32, 59–63]. The literature on the anticancer potential of CQ10 remains inconclusive. Some studies indicated that CQ10 could suppress cancer progression by degrading HIF1α, enhancing mitochondrial respiration, and inhibiting glycolysis [33–35]. Furthermore, the precursor [36] and oxidised form [37, 38] of CQ10 were shown to enhance glucose oxidation, induce ROS-mediated cell cycle arrest, and promote apoptosis in cancer cells. In HCT116 CRC cells, CQ10 also impaired growth and upregulated several tumour suppressors (e.g., p21, BAX, & p53) via increased ROS production and nitric oxide-mediated apoptosis [39]. However, other research has highlighted that CQ10 exhibited potent chemopreventive effects by reducing ROS-induced DNA damage and oncogenicity across various organs, including the colon [59–63]. Clinically, low serum CQ10 levels have been associated with higher cancer risk and poorer prognosis, likely due to reduced antioxidant capacity in tissues [30–32]. Moreover, while some studies report enhanced anticancer effects when CQ10 is combined with chemotherapies [40, 41], others indicate diminished efficacy due to its antioxidant properties [42, 43].

Therefore, we speculate that CQ10, alone and combined with either 5-FU or TQ, may increase antioxidant levels in malignant cells during the initial phase of treatment, hindering its anticancer efficacy relative to 5-FU/TQ combination [28–32, 42, 43, 59–63]. However, future studies should investigate the effects of CQ10, as single and combined therapies, on oxidative stress markers and antioxidants at different time points (e.g., 12h, 24h, & 48h) to fully elucidate its anticancer activities in CRC.

The present study is also the first to evaluate the tumoricidal effects of 5-FU/TQ/CQ10 triple therapy in CRC treatment, which exhibited the most potent anticancer effects across all cell lines tested. This was evidenced by maximal increase in apoptosis, the lowest expression of oncogenic molecules, and the highest upregulation of tumour suppressors. Additionally, the triple therapy resulted in the strongest attenuation of the PI3K/AKT/mTOR pathway, the lowest antioxidant levels, and the highest elevation in pro-oxidants relative to all other regimens.

The paradoxical roles of CQ10 in redox homeostasis may explain these boosting effects [28, 29]. CQ10 exists in three redox states: fully reduced, semi-oxidised, and fully oxidised forms, allowing it to exert opposing effects on redox biology [28, 29]. While the reduced form acts as an antioxidant, the semi-oxidised and fully oxidised forms can provoke oxidative stress by increasing electron leakage and free radical production [64–66]. Additionally, it has been shown that mitochondrial CQ10 could act as a prooxidant by increasing the generation of H^+^ ions and H_2_O_2_ through its semi-oxidised and fully oxidised forms [67, 68]. Furthermore, earlier studies have also shown that malignant cells could increase their mitochondrial CQ10 as an antioxidant defence mechanism to counteract the cytotoxic effects of chemotherapy [69, 70]. Thus, it has been suggested that increasing mitochondrial semi- and fully oxidised CQ10 could promote oxidative stress in neoplastic cells, thereby enhancing chemotherapy-induced cytotoxicity through ROS-mediated apoptosis [37, 38, 40, 41].

**Fig. 8:**
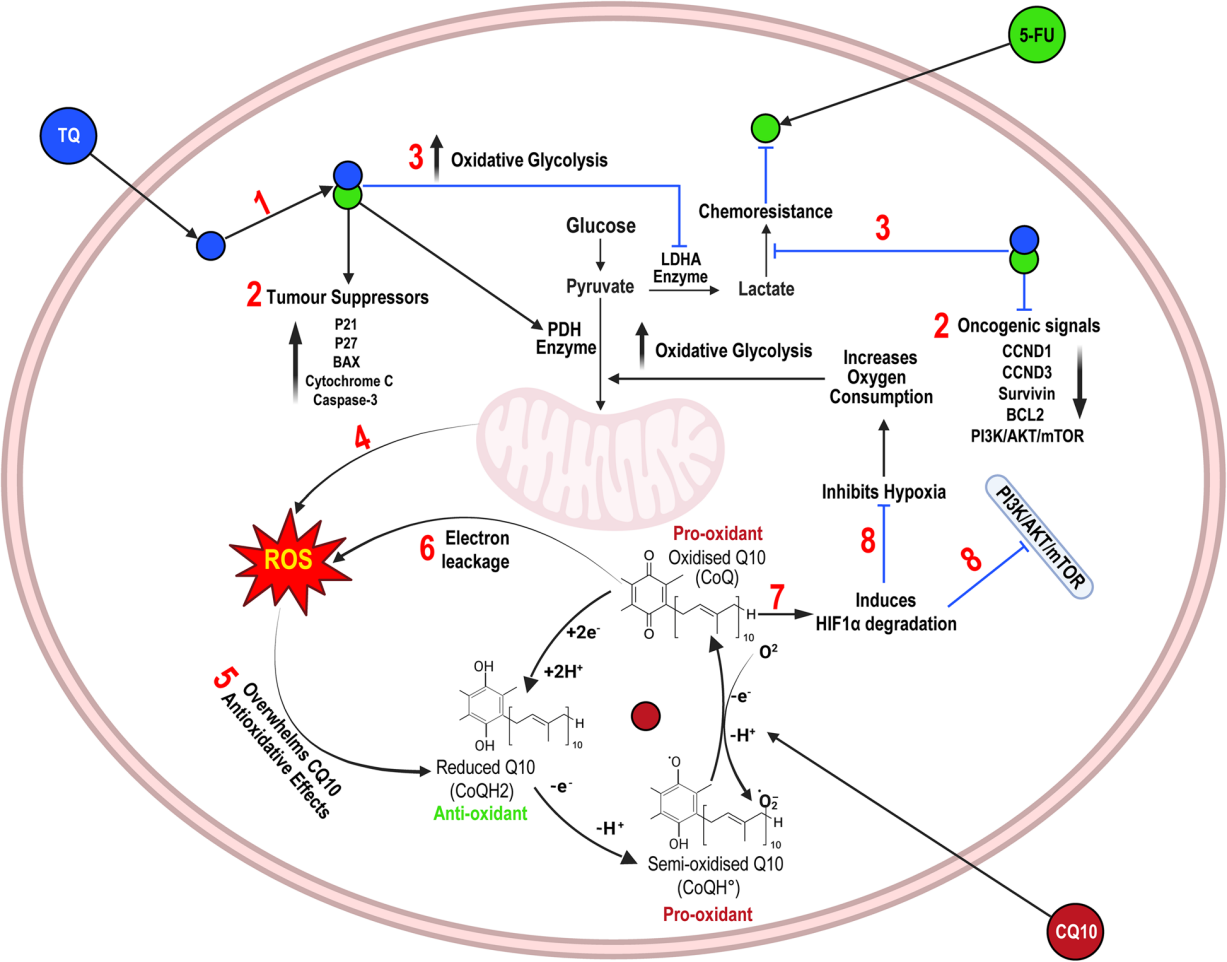
Schematic representation of the proposed mechanism of action for the 5-FU, TQ and CQ10 triple therapy in colon cancer cells. The triple therapy demonstrates synergistic anticancer effects by modulating multiple pathways. (1) TQ enhances 5-FU chemosensitivity and (2) both drugs induce apoptosis by upregulating tumour suppressors (p21, p27, BAX, Cytochrome C, Caspase-3) and inhibiting oncogenes (CCND1, CCND3, BCL2, Survivin, PI3K/AKT/mTOR). Moreover, (3) both drugs promote aerobic phosphorylation and reduce chemoresistance by inhibiting LDHA and PDHK1, while increasing PDH activity, (4) thereby elevating oxidative stress and ROS-induced cell death. CQ10, despite its antioxidant properties, acts as a pro-oxidant in this context, as (5) increased ROS levels generated by 5-FU and TQ overwhelm CQ10 antioxidant capacity, (6) leading to the generation of additional free radicals by electron leakage. Moreover, CQ10 further contributes to the anticancer effects by (7) promoting HIF1α degradation, (8) attenuating the PI3K/AKT/mTOR pathway, inhibiting hypoxia, and enhancing ROS-induced apoptosis. (Arrows: Indicate activation or increased expression; T-bars: Indicate inhibition; 5-FU: 5-Fluorouracil; AKT: protein kinase B; BAX: BCL2-associated X protein; BCL2: B-cell lymphoma 2; CCND1: cyclin D1; CCND3: cyclin D3; CQ10: coenzyme Q10; HIF1α: hypoxia-inducible factor-1α; LDHA: lactate dehydrogenase A; mTOR; mammalian target of rapamycin; p21: Cyclin-dependent kinase inhibitor 1A; p27: Cyclin-dependent kinase inhibitor 1B; PDH: pyruvate dehydrogenase; PDHK1: pyruvate dehydrogenase kinase 1; ROS, reactive oxygen species; PI3K: phosphatidylinositol-3-kinase; and TQ: thymoquinone; Figure was created by BioRender.com)

We therefore hypothesise that the superior efficacy of triple therapy could involve multiple mechanisms, including hypoxia mitigation, metabolic rewiring to oxidative glycolysis, and ROS-induced cytotoxic effects (Fig. 8). In detail, TQ may initially increase sensitivity to 5-FU, with both agents upregulating tumour suppressors, inhibiting oncogenic signals, promoting oxidative glycolysis, and increasing ROS production [25–27]. Elevated ROS levels from 5-FU and TQ could then overwhelm CQ10 antioxidative capacity, converting it to semi-oxidised and fully oxidised prooxidant forms, further aggravating oxidative stress by generating more free radicals through increased leakage of electrons [64–68]. Moreover, CQ10 may degrade HIF1α and attenuate the PI3K/AKT/mTOR pathway, thereby inhibiting hypoxia, enhancing oxidative glycolysis, and boosting the ROS-mediated tumoricidal effects of 5-FU and TQ [36–39]. However, more studies are needed to validate our proposed mechanisms (Fig. 8), particularly by measuring oxygen consumption, ATP levels, and mitochondrial ROS generation.

This study has several limitations. Firstly, the concentrations of 5-FU, TQ, and CQ10 used in this study were based on their IC50 values in vitro, which may not directly correspond to clinically achievable plasma concentrations [71, 72]. Although extrapolating results from in vitro studies to in vivo applications is a critical step in drug development and pharmacological research, it is a complex process and influenced by various factors, including bioavailability, metabolism, and tissue distribution [71, 73]. Hence, future studies should incorporate in vitro-in vivo correlation (IVIVC) approaches and animal models to establish the translatability of therapeutic doses from simple in vitro systems to more complex in vivo environments, ultimately confirming the therapeutic relevance of the drugs of interest [71–73].

We did not quantify the different CQ10 redox forms, and future research should measure the ratio of reduced/total CQ10 in isolated mitochondria to determine its role in chemoresistance [65, 66]. However, the technique is complicated and represents a significant challenge compared to measuring mitochondrial ROS production [66, 74]. Additional studies should also measure oxygen consumption, ATP production, and ROS generation under normal and hypoxic conditions to elucidate the effects of 5-FU, TQ, and/or CQ10 on mitochondrial functions in normal and malignant colon cells [22, 28, 35]. Further in vivo experiments are also necessary to evaluate the efficacy and safety of the proposed triple therapy in CRC treatment.

In conclusion, CQ10 monotherapy showed the weakest anticancer effects in all cell lines tested, likely due its potent antioxidant effects. Conversely, our data suggests that TQ monotherapy has greater proapoptotic activity than 5-FU in SW480 and SW620 cells, as indicated by stronger inhibition of oncogenic pathways, enhanced aerobic oxidation, and increased ROS levels. While statistical tests support these findings, further validation is warranted to confirm its superiority. All dual therapy groups showed higher expression of tumour suppressors and pro-apoptotic markers, elevated prooxidant levels, reduced antioxidants, and enhanced attenuation of the PI3K/AKT/mTOR pathway, with 5-FU/TQ showing superior actions to all monotherapies and the other dual therapies. On the other hand, the triple therapy displayed the best tumoricidal effects in all cell lines, suggesting a synergistic interaction involving hypoxia mitigation, oxidative glycolysis promotion, and enhanced ROS-mediated apoptosis. However, further research is needed to accurately assess the therapeutic efficacy of triple therapy against CRC, particularly by exploring mitochondrial functions related to glucose metabolism, oxygen consumption, and the ratio of reduced/total CQ10 under normal and hypoxic conditions.

## Supplementary Information

Additional file 1. Dose–response curves with IC50 values (mean ± SD) for 5-Fluorouracil (5-FU), thymoquinone (TQ), and coenzyme Q10 (CQ10) at 24h in HT29, SW480, and SW620 colon cancer cell lines, as determined using the MTT cell viability assay

Additional file 2. Cell cycle analysis data for HT29, SW480, and SW620 cells were collected for each treatment group. The proportions of cells in each phase of the cell cycle were determined from 20,000 single-cell events using the NovoExpress cell cycle algorithm, which calculates the percentage of cells in each phase (histograms). The plots presented are representative of one of three similar experiments, and the percentages of cells in each phase are shown as mean ± SD (*n* = 3).

Additional file 3. Apoptosis analysis data for HT29, SW480, and SW620 cells were collected for each treatment group. The proportions of living, early apoptotic, late apoptotic, and dead cells were determined from 20,000 single-cell events using an Acea Novocyte 3000 flow cytometer, following staining with the Annexin V-FITC/PI Apoptosis Assay Kit. The scatter plots presented are representative of one of three similar experiments, and the percentages of live (AV-/PI-), early apoptotic (AV + /PI-), late apoptotic (AV + /PI +), and dead (AV-/PI +) cells are shown as mean ± SD (*n* = 3).

Additional file 4.

## Data Availability

No datasets were generated or analysed during the current study.
