# Supplementary figures and images for: Exploring potential additive effects of 5-fluorouracil, thymoquinone, and coenzyme Q10 triple therapy on colon cancer cells in relation to glycolysis and redox status modulation
Source: J Egypt Natl Canc Inst. 2025 Mar 10;37:7. doi: 10.1186/s43046-025-00261-7 (PMC13313436; doi:10.1186/s43046-025-00261-7)

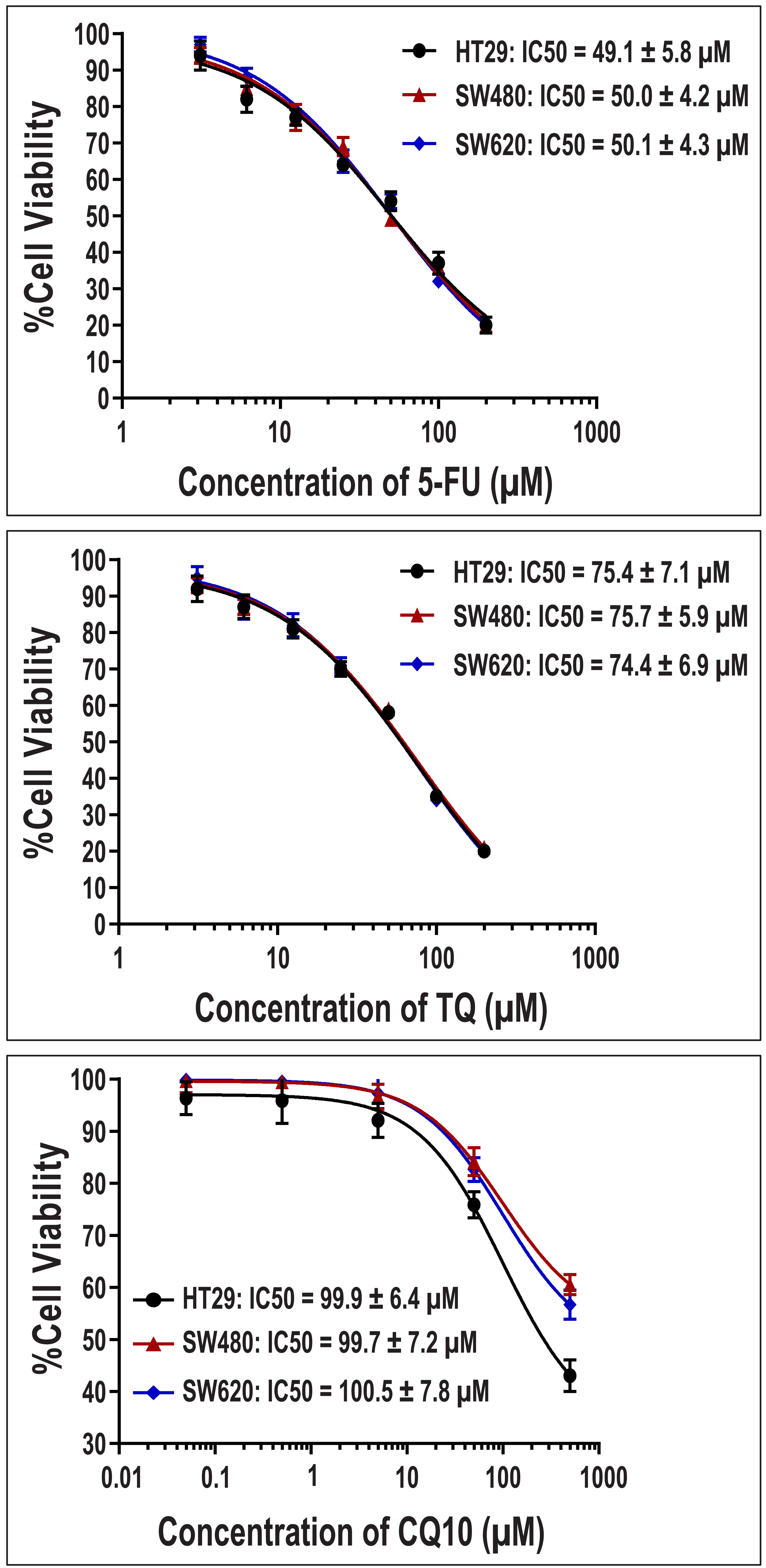

Supplement: Supplementary file 1 — Additional file 1. Dose–response curves with IC50 values (mean ± SD) for 5-Fluorouracil (5-FU), thymoquinone (TQ), and coenzyme Q10 (CQ10) at 24h in HT29, SW480, and SW620 colon cancer cell lines, as determined using the MTT cell viability assay [file 43046_2025_261_MOESM1_ESM.tif]

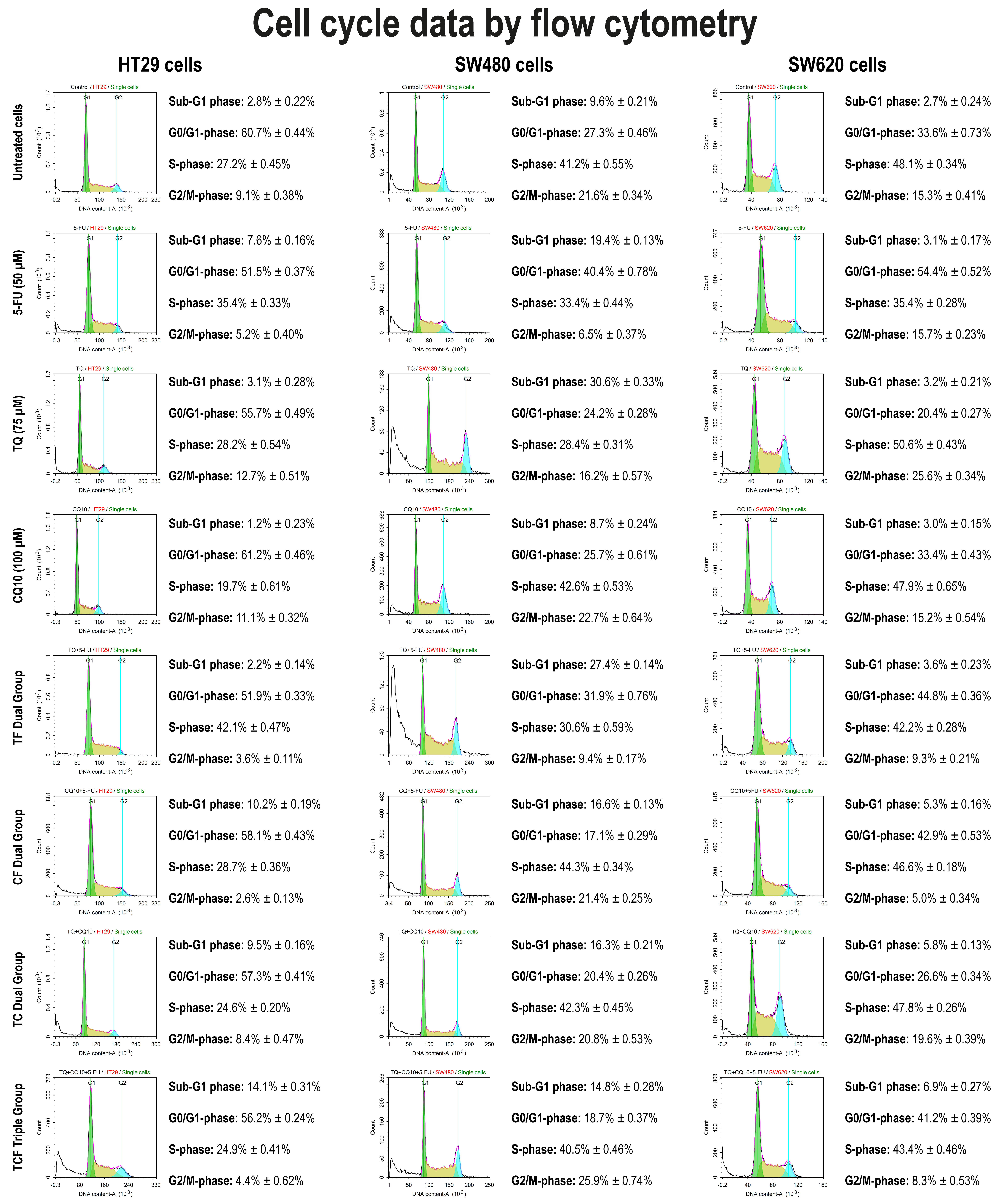

Supplement: Supplementary file 2 — Additional file 2. Cell cycle analysis data for HT29, SW480, and SW620 cells were collected for each treatment group. The proportions of cells in each phase of the cell cycle were determined from 20,000 single-cell events using the NovoExpress cell cycle algorithm, which calculates the percentage of cells in each phase (histograms). The plots presented are representative of one of three similar experiments, and the percentages of cells in each phase are shown as mean ± SD (n = 3). [file 43046_2025_261_MOESM2_ESM.tif]

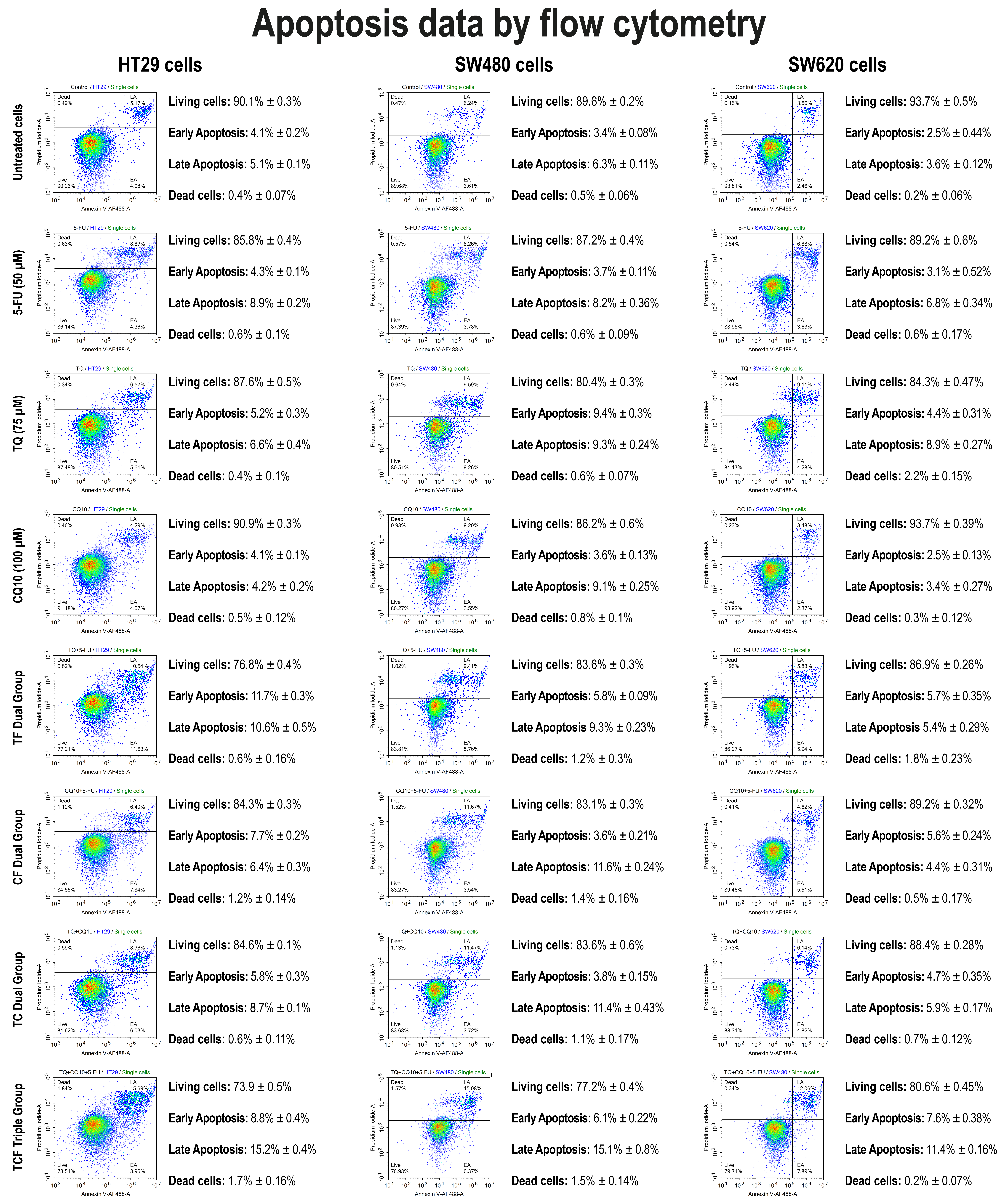

Supplement: Supplementary file 3 — Additional file 3. Apoptosis analysis data for HT29, SW480, and SW620 cells were collected for each treatment group. The proportions of living, early apoptotic, late apoptotic, and dead cells were determined from 20,000 single-cell events using an Acea Novocyte 3000 flow cytometer, following staining with the Annexin V-FITC/PI Apoptosis Assay Kit. The scatter plots presented are representative of one of three similar experiments, and the percentages of live (AV-/PI-), early apoptotic (AV + /PI-), late apoptotic (AV + /PI +), and dead (AV-/PI +) cells are shown as mean ± SD (n = 3). [file 43046_2025_261_MOESM3_ESM.tif]
