# Supplementary material for: Exploring potential additive effects of 5-fluorouracil, thymoquinone, and coenzyme Q10 triple therapy on colon cancer cells in relation to glycolysis and redox status modulation
Source: J Egypt Natl Canc Inst. 2025 Mar 10;37:7. doi: 10.1186/s43046-025-00261-7 (PMC13313436; doi:10.1186/s43046-025-00261-7)
Supplement: Supplementary file 4 — Additional file 4. [file 43046_2025_261_MOESM4_ESM.docx]

**Supplementary table 1.** Sequences of PCR primers used for detecting human *GAPDH*, *PI3KCA, AKT1, mTOR, RICTOR, RAPTOR, PTEN, CCND1, CCND3, CDKN1A, CDKN1B, BCL2, Survivin, Cytochrome C, BAX, and Caspase-3* mRNAs in human colon cancer cells including their corresponding gene accession numbers and amplicon sizes.

| **Genes** | **Forward** | **Reverse** | **Amplicon size** |
| --- | --- | --- | --- |
| ***GAPDH***  (NCBI: NM_002046.5) | 5’ CAC ATG GCC TCC AAG GAG TAA 3’ | 5’ TGA GGG TCT CTC TCT TCC TCT TGT 3’ | 74 bp |
| ***PIK3CA***  (NCBI: NM_006218.4) | 5’ GGA CCC GAT GCG GTT AGA G 3’ | 5’ ATC AAG TGG ATG CCC CAC AG 3’ | 168 bp |
| ***AKT1***  (NCBI: NM_001382431.1) | 5’ CTC AGT GTC GTC AGA GCC C 3’ | 5’ ATG GAA AGC AGG CCA GAC TC 3’ | 100 bp |
| ***mTOR***  (NCBI: NM_004958.4) | 5’ GAC GAG AGA TCA TCC GCC AG 3’ | 5’ ACA AGG GAC CGC ACC ATA AG 3’ | 97 bp |
| ***PTEN***  (NCBI: NM_000314.8) | 5’ CTC AGC CGT TAC CTG TGT GT 3’ | 5’ AGG TTT CCT CTG GTC CTG GT 3’ | 129 bp |
| ***mTOR***  (NCBI: NM_004958.4) | 5’ GAC GAG AGA TCA TCC GCC AG 3’ | 5’ ACA AGG GAC CGC ACC ATA AG 3’ | 97 bp |
| ***RICTOR***  (NCBI: NM_152756.5) | 5’ GCT GGA TCT GAC CCG AGA AC 3’ | 5’ CCC AGT TTT TCT TCA CTG TGG C 3’ | 153 bp |
| ***RAPTOR***  (NCBI: NM_020761.3) | 5’ CGG ACC TCG TGA AGG ACA AC 3’ | 5’ TGA CGA TCA CGG CGA GAA TG 3’ | 111 bp |
| ***PTEN***  (NCBI: NM_000314.8) | 5’ CTC AGC CGT TAC CTG TGT GT 3’ | 5’ AGG TTT CCT CTG GTC CTG GT 3’ | 129 bp |
| ***CCND1***  (NCBI: NM_053056.2) | 5’ TGA CCC CGC ACG ATT TCA TT 3’ | 5’ CAT GGA GGG CGG ATT GGA AA 3’ | 143 bp |
| ***CCND3***  (NCBI: NM_001136017.3) | 5’ GGT GCA ATC CTC TCC TCG C 3’ | 5’ TAG TTC ATG GCC AGG GGG AA 3’ | 183 bp |
| ***CDKN1A***  (NCBI NM_000389.4) | 5’ AGT CAG TTC CTT GTG GAG CC 3’ | 5’ GCA TGG GTT CTG ACG GAC AT 3’ | 109 bp |
| ***CDKN1B***  (NCBI: NM_004064.4) | 5’ CTG GCC TCA GAA GAC GTC AAA 3’ | 5’ AGG ATG TCC ATT CCA TGA AGT CAG 3’ | 147 bp |
| ***BCL2***  (NCBI: NM_000633.3) | 5’ TTT CGG TGA CTT CCG CAT CA 3’ | 5’ CGG TCT CCT AAA AGC AGG CA 3’ | 79 bp |
| ***Survivin***  (NCBI: NM_001168.2) | 5’ CCA CTG AGA ACG AGC CAG AC 3’ | 5’ GCA ACC GGA CGA ATG CTT TT 3’ | 113 bp |
| ***Cytochrome C***  (NCBI: NM_018947.6) | 5’ CGT TGT GCC AGC GAC TAA AA 3’ | 5’ TGG CAC TGG GAA CAC TTC AT 3’ | 88 bp |
| ***BAX***  (NCBI: NM_001291428.1) | 5’ TCG CCC TTT TCT ACT TTG CCA 3’ | 5’ GTC CTG GAG ACA GGG ACA TCA 3’ | 195 bp |
| ***CASP3***  (NCBI: NM_004346.3) | 5’ CTC TGG TTT TCG GTG GGT GT 3’ | 5’ CCA CTG AGT TTT CAG TGT TCT CC 3’ | 90 bp |
